# Genes located in Y-chromosomal regions important for male fertility show altered transcript levels in cryptorchidism and respond to curative hormone treatment

**DOI:** 10.1186/s12610-019-0089-3

**Published:** 2019-06-03

**Authors:** Katharina Gegenschatz-Schmid, Gilvydas Verkauskas, Michael B. Stadler, Faruk Hadziselimovic

**Affiliations:** 1 Cryptorchidism Research Institute, Kindermedizinisches Zentrum Liestal, 4410 Liestal, Switzerland; 2 Children’s Surgery Centre, Faculty of Medicine, Vilnius of University, 01513 Vilnius, Lithuania; 3 Friedrich Miescher Institute for Biomedical Research, Basel, Switzerland; 4 Swiss Institute of Bioinformatics, Basel, Switzerland

**Keywords:** Y-chromosome, AZF region, Ad spermatogonia, RNA-sequencing, Testosterone, LH, GnRHa treatment, Infertility, Cryptorchidism, Mini-puberty, Chromosome Y, Région AZF, Spermatogonie Ad, Séquençage des ARN, Testostérone, LH, Traitement par GnRHa, Infertilité, Cryptorchidie, Mini-puberté

## Abstract

**Background:**

Undescended (cryptorchid) testes in patients with defective mini-puberty and low testosterone levels contain gonocytes that fail to differentiate normally, which impairs the development of Ad spermatogonia and ultimately leads to adult infertility. Treatment with the gonadotropin-releasing hormone agonist GnRHa increases luteinizing hormone and testosterone and rescues fertility in the majority of pathological cryptorchid testes. Several Y-chromosomal genes in the male-specific Y region (MSY) are essential for spermatogenesis, testis development and function, and are associated with azoospermia, infertility and cryptorchidism. In this study, we analyzed the expression of MSY genes in testes with Ad spermatogonia (low infertility risk patients) as compared to testes lacking Ad spermatogonia (high infertility risk) before and after curative GnRHa treatment, and in correlation to their location on the Y-chromosome.

**Results:**

Twenty genes that are up- or down-regulated in the Ad- group are in the X-degenerate or the ampliconic region, respectively. GnRHa treatment increases mRNA levels of 14 genes in the ampliconic region and decreases mRNA levels of 10 genes in the X-degenerate region.

**Conclusion:**

Our findings implicate Y-chromosomal genes, including *USP9Y, UTY, TXLNGY, RBMY1B, RBMY1E, RBMY1J and TSPY4*, some of which are known to be important for spermatogenesis, in the curative hormonal treatment of cryptorchidism-induced infertility.

## Résumé

**Contexte:**

La non descente des testicules chez les garçons cryptorchides qui présentent une mini-puberté défectueuse et un taux réduit de testostérone (T) ont des gonocytes incapables de se différencier normalement en spermatogonie Ad. Cette dernière entraîne une infertilité. Le traitement avec l’agoniste du GnRH (GnRHa) augmente les taux de LH et T et permet de sauvegarder la fertilité chez la majorité des testicules cryptorchides pathologiques. Plusieurs gènes du chromosome Y localisés dans la région spécifique du mâle (MSY) sont essentiels pour la spermatogénèse, ainsi que pour le développement et la fonction testiculaires, et sont associés à l’azoospermie, l’infertilité et la cryptorchidie. Dans cette étude, nous avons analysé l’expression des gènes dans la région MSY des testicules avec et sans spermatogonies Ad, avant et après traitement par GnRHa. Les résultats sont corrélés avec la localisation des gènes dans le chromosome Y.

**Résultats:**

Dans le groupe Ad-, vingte gènes dont l’expression est. régulée à la hausse ou à la baisse sont respectivement localisés dans la région dégénérée du X ou dans la région ampliconique. Le traitement par GnRHa augmente les taux de mRNA de 14 gènes dans la région ampliconique et réduit l’expression de 10 gènes dans la région dégénérée du X.

**Conclusion:**

Nos résultats impliquent une participation des gènes du chromosome Y, compris *USP9Y, UTY, TXLNGY, RBMY1B, RBMY1E, RBMY1J et TSPY4*, dont certains sont importants pour la fertilité, dans le traitement curatif de l’infertilité due à la cryptorchidie.

## Introduction

Cryptorchidism is the most frequent congenital pediatric urological disorder in boys and represents the most common cause of non-obstructive azoospermia in man [1–3]. During mini-puberty, which peaks between 30 to 60 days and lasts up to 180 days of postnatal life in male infants, activation of the hypothalamic-pituitary-gonadal (HPG) axis leads to a transient increase of gonadotropins and testosterone [4–6], which induce the transition of gonocytes into Ad (dark) spermatogonia that are stem cells for sperm development [7, 8]. In cryptorchid testes with defective mini-puberty, insufficient testosterone levels fail to direct gonocytes into the differentiation process, which impairs the development of Ad spermatogonia and ultimately causes adult infertility [9–11]. Treatment with the gonadotropin-releasing hormone agonist (GnRHa) Buserelin increases luteinizing hormone (LH) and testosterone levels and rescues fertility in the majority of cryptorchid boys [12]. We reported earlier that GnRHa induces expression of genes important for the HPG axis [8, 13] and the gonocyte-Ad spermatogonia transition [14], and has a repressive effect on Sertoli cell marker genes [15].Someof these reported GnRHa regulated genes, are localized on the Y chromosome.

**Fig. 1:**
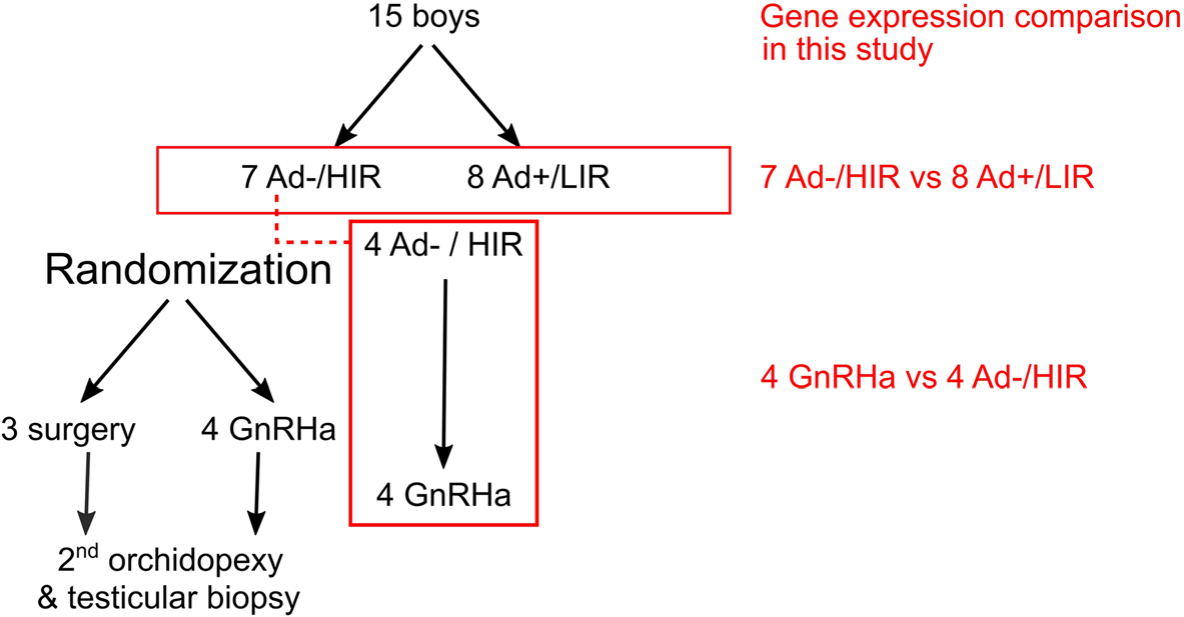
Flow chart showing study design, and the selection of study patients and samples for RNA expression profiling

The Y chromosome harbors a number of genes essential for spermatogenesis, testis development and function, which are located in the male-specific Y region (MSY), known as non-recombining region of the Y chromosome ([16] and reviewed in [17]). The euchromatic sequences of the MSY have been divided into three classes on the basis of their evolutionary origin [18]: X-transposed, X-degenerate and ampliconic (Fig. 1). Interestingly, ubiquitously expressed genes were found to reside in X-degenerate regions, while exclusively testes specific protein coding genes were found in the ampliconic regions [18]. Especially deletions on the long arm of the Y chromosome (Yq) were associated with defects in spermatogenesis and are designated as azoospermia factor (AZF) regions [16, 19]. Based on particular spermatogenesis disruption phenotypes, three AZF regions were defined: (1) AZFa deletions were associated with complete absence of germ cells in tubules. (2) AZFb deletions were associated with a maturation arrest at the spermatocyte stage. (3) AZFc deletions were associated with hypospermatogenesis [16], reviewed in [20]. The AZFa region contains three protein coding genes *DDX3Y*, *USP9Y*, *UTY* and the long non-coding RNA (lncRNA) *TTTY15*, and deletions are frequently observed in Sertoli cell-only (SCO) syndrome [21–23]. *UTY* belongs to the group of H3K27me2/3 histone demethylases, which are involved in male germ cell maintenance and development [24–26]. AZFb and AZFc deletions partially overlap. Male specific RBMY proteins are predominantly expressed in post-meiotic germ cells and bind RNA [27–29]. Both RBMY and the lysine-specific histone (H3K4) demethylase KDM5D are considered candidates for causing AZFb-related testicular pathology; reviewed in [20]. The AZFc region is almost exclusively constituted by amplicons and contains three gene families (*BPY2*, *CDY* and *DAZ*) and the lncRNAs *TTTY3* and *TTTY4*. CDY proteins are histone acetyltransferases with a strong preference for H4 and are considered as nuclear remodeling factors promoting histone H4 hyperacetylation in late spermatids [30]. Deletion of *DAZ* genes are common causes of infertility in humans. DAZ family members are RNA binding proteins important in the establishment and maintenance of the male germ line; reviewed in [31–33]. Genetic mapping of the short arm of the Y chromosome (Yp) resulted in the localization of the sex-determining gene *SRY* [34, 35] and the gonadoblastoma (GBY) locus with *TSPY* as the putative gene locus [36, 37].

In this study, we investigated the expression of male-specific Y chromosomal genes in undescended testis prone to infertility by comparing RNA profiles from testes with impaired mini-puberty lacking Ad spermatogonia (High Infertility Risk, Ad-) to those from testes that completed mini-puberty (Low Infertility Risk, Ad+). Furthermore, we analyzed the effect of GnRHa on MSY gene expression in Ad- patients. Our results implicate Y-chromosome genes important for spermatogenesis in the curative hormonal treatment of cryptorchidism-induced infertility.

## Materials and methods

### Study population and biopsy sample collection

Testis localized outside of the scrotum and incapable of being brought into a stable scrotal position is defined as a cryptorchid testis. In our earlier studies all patients with isolated cryptorchidism had undescended testes located in the inguinal region [8, 13]. Patients were age and ethnicity matched. The age of the patients ranged from 8 to 59 months, resulting in a median age of 18.5 months. Testicular biopsies were taken at the time of orchidopexy. Collected biopsy samples were divided into two pieces, with one fragment immediately immersed in RNAlater (ThermoFisher Scientific, Waltham, Massachusetts, USA) and stored at − 25 °C until further processing (for RNA extraction and RNA- sequencing), and the other fixed in glutaraldehyde for histological processing.

To evaluate Y-chromosomal gene expression profiles we used RNA sequencing data from our two previous studies: The first study included 15 biopsies of 15 patients (7 unilateral and 8 bilateral undescended testes) which were selected prior randomization and based on histological results (Fig. 1). Seven patients were grouped into the High Infertility Risk group lacking Ad spermatogonia (HIR/Ad-), and 8 patients were grouped into the Low Infertility Risk group presenting Ad spermatogonia (LIR/Ad+) [8]. From a randomized study [38], in which Ad- bilateral cryptorchid boys were treated with GnRHa (Buserelin) after the first orchidopexy (surgery), data was retrieved from 4 patients. Initial biopsies of these four patients revealed no Ad spermatogonia, indicating defective mini-puberty (Ad- group). The second testis was managed by orchidopexy and biopsied 6 months after the initial surgery and GnRHa treatment [13]. Since data of first biopsies of two out of these four patients was retrieved from the HIR(Ad-)/LIR(Ad+) comparison study (15 biopsies), in total results from 21 biopsies were compared.

### Histological analyses

Biopsies were fixed in phosphate-buffered saline (PBS, pH 7.4) containing 3% glutaraldehyde and embedded in Epon resin. Semi-thin sections of 1 μm were cut using a Reichert Om-U3 ultramicrotome (Reichert AG, Vienna, Austria). Sections were mounted on glass slides, stained with 1% toluidine blue, and examined under a Zeiss Axioskop light microscope (Carl Zeiss Microscopy GmbH, Jena, Germany) with an integrated photo-camera.

During histological analyses, at least 100 tubular cross sections per biopsy were evaluated, regarding their number of Ad spermatogonia. Ad spermatogonia were identified in prepubertal testes according to the criteria first published by Seguchi and Hadziselimovic [39]. Ad spermatogonia are germ cells, which in contrast to Ap or fetal spermatogonia, are characterized by cytoplasm with a darker aspect and a typical halo in the nucleus, termed the rarefaction zone.

### RNA preparation, sequencing, data analyses, and RNA expression levels

The workflow from RNA isolation, through to purification, library preparation, sequencing, data analyses, and expression level analysis, was described earlier in detail [8, 13].

### Data and differential gene expression analyses

Determination of differentially expressed genes, statistical analyses and model design were described previously [8, 13]. Only genes with at least one read per million, in at least two samples, were included. *P* values and fold-changes were calculated for the treatment factor and differentially expressed genes were defined as those displaying a false discovery rate (FDR) of less than 0.05. Raw data files are deposited at the Database of Genotypes and Phenotypes (dbGaP) with the accession number phs001275.v1.p1.

## Results

**Fig. 2:**
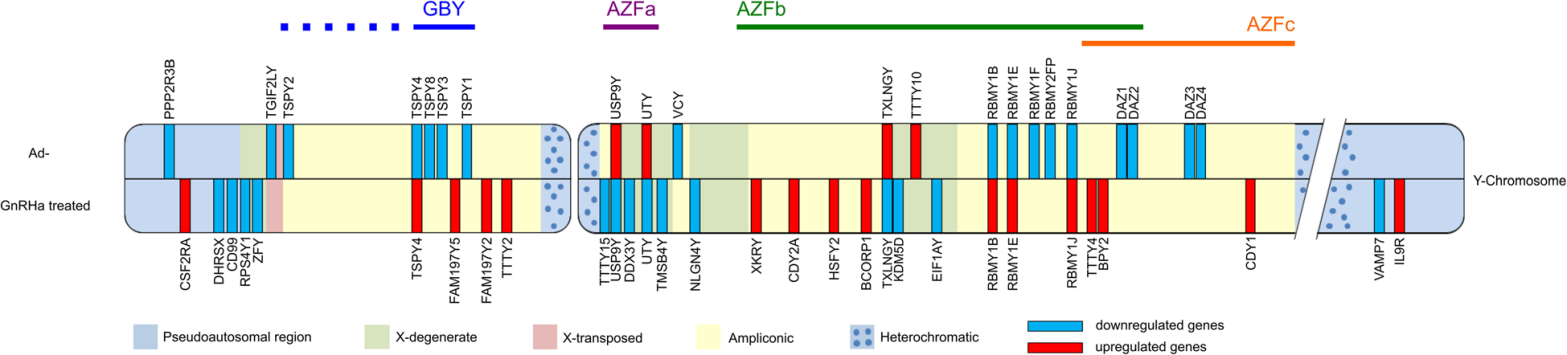
Illustration of Y-chromosomal differential gene expression in Ad- versus Ad+ testis and Ad- testis before and after GnRHa treatment. Features of the Y-chromosome are represented as colored blocks and include the pseudo-autosomal region (blue), heterochromatic (dotted blue), X-degenerate (green), ampliconic (yellow) and X-transposed (red) regions. The upper half of the Y-chromosome shows differentially expressed genes observed in Ad- testes and the lower half differentially expressed genes in GnRHa testes. Genes for which we measured increased or decreased mRNA levels are given in blue or red, respectively. Azoospermia factor regions (AZFa-c) and gonadoblastoma locus on Y chromosome (GBY) are indicated

We recently reported the differential gene expression profiles of Ad- versus Ad+ and GnRHa treated versus untreated Ad- patients [8, 13], of which 10 genes are of Y chromosomal origin (Fig. 2). This let us in this study, to focus on 577 genes mapped on the Y chromosome (RefSeq genome records for *Homo sapiens*, annotation release 108). We found 10 additional genes (20 in total) that are significantly differentially expressed between Ad- and Ad+ samples (Tables 1 and 2). Furthermore, we identified 21 additional (25 in total) differentially expressed genes when we compared GnRHa treated and untreated Ad- patient samples, all of which showed significant differences (Tables 1 and 2). For clarity, this analysis focusses on protein-coding and non-coding genes in the MSY region, excluding the Y-chromosomal pseudoautosomal and recombining regions.

**Table 1:**
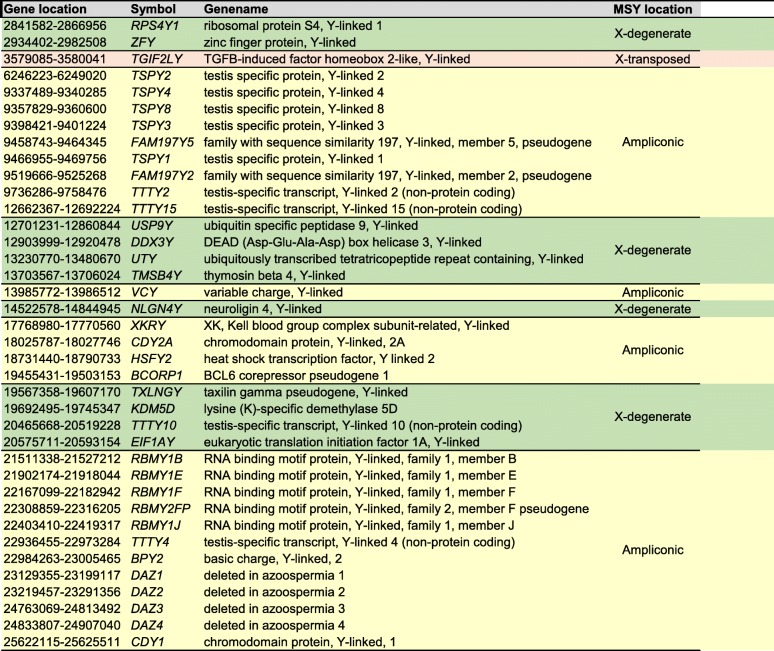
List of male specific Y-chromosomal (MSY) genes analyzed in this study. Gene annotation (Symbol), full genename, and gene location in the MSY region (X-degenerate, X-transposed, or ampliconic, highlighted in green, red and yellow, respectively) are represented

**Table 2:**
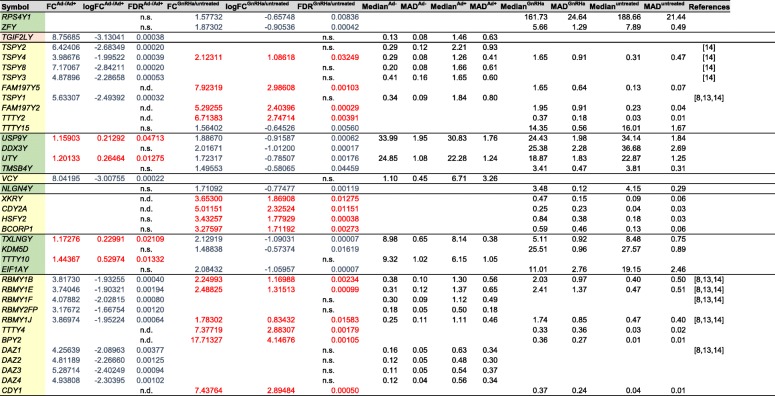
Differential expression of male specific Y-chromosomal (MSY) genes in Ad- versus Ad+ and in the GnRHa treated versus untreated Ad- groups (GnRHa/untreated). Genes are ordered according to their location on the Y chromosome. Increased or decreased gene expression is displayed in red or blue values, respectively. The table contains information on the location of the gene in the MSY region colored as described in Table 1, the log-transformed fold change (log FC^Ad−/Ad+^), false discovery rate (FDR^Ad−/Ad+^), median expression values in reads per kilobase per million (RPKM) (Median^Ad-^; Median^Ad+^), and the median absolute deviation (MAD^Ad-^; MAD^Ad+^) for Ad- and Ad+ samples. A similar nomenclature is applied for comparison of Ad- samples before and after treatment (GnRHa/untreated). Values showing differences that are not significant (n.s.) or not determined (n.d.) are indicated. Earlier reported genes are listed with the corresponding reference

### Genes that are up- or down-regulated in the ad- group are in the X-degenerate or the ampliconic region, respectively

*USP9Y*, *UTY*, *TXLNGY* and *TTTY10* are in the X-degenerate region and show slightly increased mRNA levels in the Ad- group as compared to the Ad+ group (Tables 1 and 2).

As opposed to that, 16 genes showed decreased mRNAs levels in the Ad- group compared to the Ad+ group. Except for *TGIF2LY*, which is found in the X-transposed region, the downregulated genes are located in the ampliconic region (Tables 1 and 2, Fig. 1). These loci include the deleted in azoospermia family genes *DAZ1*, *DAZ2*, *DAZ3*, *DAZ4*, the Y-linked testis specific protein coding family genes *TSPY1*, *TSPY2*, *TSPY3*, *TSPY4*, *TSPY8,* the RNA binding motif protein Y-linked family 1 members *RBMY1B*, *RBMY1E*, *RBMY1F*, *RBMY2FP*, *RBMY1J*, and finally the Y-linked variable charge gene *VCY*.

### GnRHa treatment increases mRNA levels of genes in the ampliconic region and decreases mRNA levels of genes in the X-degenerate region

Eleven genes within the MSY showed decreased mRNA levels in testes from Ad- patients after GnRHa treatment (Tables 1 and 2, Fig. 1). Except for *TTTY15*, which is in the ampliconic region, they are located in the X-degenerate region (Tables 1 and 2, Fig. 1): *DDX3Y*, *EIF1AY*, *KDM5D*, *NLGN4Y*, *RPS4Y1*, *TMSB4Y*, *TXLNGY*, *USP9Y*, *UTY*, and *ZFY*.

Fourteen genes are upregulated in samples from Ad- patients after GnRHa treatment and are in the ampliconic region (Tables 1 and 2, Fig. 1): *BCORP1*, *BPY2*, *CDY1*, *CDY2A*, *FAM197Y2*, *FAM197Y5*, *HSFY2*, *RBMY family members 1B, −1E, and -1 J*, *TSPY4*, *TTTY2*, *TTTY4*, and *XKRY*.

### USP9Y, UTY and TXLNGY show elevated mRNA levels in ad- samples and negatively respond to GnRHa treatment

Three genes show reduced RNA expression levels in Ad- patient samples and increased RNA levels after GnRHa treatment (Table 2): *USP9Y*, *UTY*, and *TXLNGY*. The genes are located within the AZF deletion regions (Fig. 1).

### RBMY1B, RBMY1E, RBMY1J and TSPY4 show reduced mRNA levels in ad- samples and positively respond to GnRHa treatment

Four genes show reduced RNA expression levels in Ad- patient samples and increased RNA levels after GnRHa treatment (Table 2): *RBMY1B*, *RBMY1E*, *RBMY1J*, and *TSPY4*. The genes are located within the ampliconic deletion regions (Fig. 1).

## Discussion

During mini-puberty GnRH induces differentiation of Ad spermatogonia from gonocytes. Treatment with GnRHa in cryptorchid boys of the HIR group (Ad-) was effective in rescuing defective mini-puberty and completing the transition from gonocytes to Ad spermatogonia [38]. The differential gene expression results of Y chromosome genes suggest transcriptional changes during mini-puberty, supporting the differentiation process of Ad spermatogonia from gonocytes and suggesting GnRHa dependent responsiveness especially for *USP9Y, UTY, TXLNGY, RBMY1B, RBMY1E, RBMY1J and TSPY4*.

The Y chromosome harbors a number of genes important for male fertility. We find that positive and negative effects of cryptorchidism and curative hormonal treatment of gonocyte differentiation appear to be concentrated in defined chromosomal regions (Fig. 1).

What might be the mechanism for such broad and region-specific effects on gene expression? The epigenetic pattern on the human Y chromosome was found to be evolutionary conserved [37]. It was shown that the DNA methylation pattern was relatively stable compared to the tested X chromosome and chromosome 12 [37]. Furthermore, Singh and coworkers observed that the global conservation of the epigenetic pattern was associated with sequences of the same origin (X-transposed, X-degenerate, ampliconic), implying similar regulatory mechanisms across genes that share common origin and epigenetic profile [40].

Epigenetically regulated gene expression during spermatogenesis is critical for development of fertility. During the different steps of spermatogenesis, several epigenetic modifications involving DNA methylations and histone modifications occur; reviewed in [41]. While primordial germ cells undergo a process of demethylation and deacetylation, a progressive DNA methylation occurs in spermatogonia with establishment of paternal methylation. Several studies reported epigenetic changes as cause for infertility in men, including altered methylation of various imprinted and developmental loci [42–45], and abnormal histone marks [46, 47]. Although, to our best knowledge, no specific DNA methylation changes on the Y chromosome have been linked to infertility, they have been connected to prostate cancer [48]. GnRHa treatment had a gene repressing effect on *UTY* and *KDM5D*, both of which are demethylases of the repressing mark Histone H3 Lysine 27 (H3K27me3) [49] and the activating mark Histone H3 Lysine 4 (H3K4me3) [50, 51], respectively. *UTY* is thought to have lost its histone demethylase activity but the gene was shown to be important for mouse embryogenesis independently of demethylase enzyme activity [52]. It is therefore possible that this new function also operates in human gonocytes, and GnRHa treatment influences histone modifications.

Little is known about the functions of *TSPY4* and *TXLNGY* in human and there are no known mouse homologs. *USP9Y* was initially implicated in male fertility but later it was found that the gene was deleted in patients with normal spermatogenesis, which argues against a critical function in the process [53]. Foresta and coworkers suggested that *DBY/DDX3Y* might be an AZFa candidate because it is frequently deleted in male infertility, and its mutation significantly reduces or even abolishes the germ cell population [54]. GnRH treatment greatly downregulated *DBY/DDX3Y* expression, indicating that full level expression of this gene is not essential for gonocyte differentiation into Ad spermatogonia. *RBMY* is critical for male fertility in a mouse model and therefore constitutes a major candidate for molecular functions that may help explain the curative effect of GnRHa treatment [55]. While the limitation of this exploratory Y-chromosomal RNA profiling study is the small number of samples, we would like to point out that the included patients were enrolled sequentially and received treatment based on a randomized allocation (Fig. 2) [38].

## Conclusion

Our findings link Y-chromosomal genes known to be important and relevant for spermatogenesis in the curative hormonal treatment of cryptorchidism-induced infertility. Of note, our observation support data of global conservations of the epigenetic pattern associated with the sequences of the same origin (X-transposed, X-degenerate and ampliconic). This observations implicate Y-chromosomal genes, including *USP9Y, UTY, TXLNGY, RBMY1B, RBMY1E, RBMY1J and TSPY4*, some of which are known to be important for spermatogenesis, in the curative hormonal treatment of cryptorchidism-induced infertility.

## Abbreviations

AZF: Azoospermia Factor
AZFa: Azoospermia Factor region a
AZFb: Azoospermia Factor region b
AZFc: Azoospermia factor region c
BCORP1: BCL6 corepressor pseudogene 1
BPY: Basic charge, Y-linked, 2
CDY: Chromodomain protein, Y-linked,
DAZ: Deleted in azoospermia
dbGaP: Database of Genotypes and Phenotypes
DBY/DDX3Y: DEAD-Box Helicase 3 Y-Linked
EIF1AY: Eukaryotic translation initiation factor 1A, Y-linked
FAM197Y: Family with sequence similarity 197, Y-linked
FDR: False discovery rate
GBY: Gonadoblastoma Y-locus
GnRHa: Gonadotropin-releasing hormone agonist
H3K27me3: Trimethylated histone H3 protein of lysine 27
H3K4me3: Trimethylated histone H3 protein of lysine 4
HIR/Ad-: High Infertility Risk group lacking Ad spermatogonia
HPG: Hypothalamic-pituitary-gonadal
HSFY2: Heat shock transcription factor, Y linked 2
KDM5D: Lysine (K)-specific demethylase 5D
LH: Luteinizing hormone
LIR/Ad+: Low Infertility Risk group presenting Ad spermatogonia
lncRNA: Long non-coding RNA
MSY: Male-specific Y region
NLGN4Y: Neuroligin 4, Y-linked
PBS: Phosphate-buffered saline
RBMY: RNA binding motif protein, Y-linked
RBMY2FP: RNA binding motif protein, Y-linked, family 2, member F pseudogene
RPS4Y1: Ribosomal protein S4, Y-linked 1
SCO: Sertoli cell-only
SRY: Sex determining region of Y
TGIF2LY: TGFB-induced factor homeobox 2-like, Y-linked
TSPY: Testis-specific protein, Y-linked
TTTY: Testis-specific transcript, Y-linked
TXLNGY: Taxilin gamma pseudogene, Y-linked
USP9Y: Ubiquitin specific peptidase 9, Y-linked
UTY: Ubiquitously transcribed tetratricopeptide repeat containing, Y-linked
VCY: Variable charge, Y-linked
XKRY: XK, Kell blood group complex subunit-related, Y-linked
ZFY: Zinc finger protein, Y-linked
